# IFITM3 Inhibits SARS-CoV-2 Infection and Is Associated with COVID-19 Susceptibility

**DOI:** 10.3390/v14112553

**Published:** 2022-11-18

**Authors:** Fengwen Xu, Geng Wang, Fei Zhao, Yu Huang, Zhangling Fan, Shan Mei, Yu Xie, Liang Wei, Yamei Hu, Conghui Wang, Shan Cen, Chen Liang, Lili Ren, Fei Guo, Jianwei Wang

**Affiliations:** 1 NHC Key Laboratory of Systems Biology of Pathogens, Institute of Pathogen Biology, and Center for AIDS Research, Chinese Academy of Medical Sciences & Peking Union Medical College, Beijing 100730, China; 2 NHC Key Laboratory of Systems Biology of Pathogens and Christophe Mérieux Laboratory, Institute of Pathogen Biology, Chinese Academy of Medical Sciences & Peking Union Medical College, Beijing 100730, China; 3 Department of Respiratory and Critical Care Medicine, Clinical Research Center for Respiratory Disease, West China Hospital, Sichuan University, Chengdu 610041, China; 4 Institute of Medicinal Biotechnology, Chinese Academy of Medical Sciences & Peking Union Medical College, Beijing 100050, China; 5 Lady Davis Institute, Jewish General Hospital, McGill University, Montreal, QC H3T 1E2, Canada; 6 Key Laboratory of Respiratory Disease Pathogenomics, Chinese Academy of Medical Sciences and Peking Union Medical College, Beijing 100730, China

**Keywords:** SARS-CoV-2, COVID-19, IFITM3, rs12252

## Abstract

SARS-CoV-2 has become a global threat to public health. Infected individuals can be asymptomatic or develop mild to severe symptoms, including pneumonia, respiratory distress, and death. This wide spectrum of clinical presentations of SARS-CoV-2 infection is believed in part due to the polymorphisms of key genetic factors in the population. In this study, we report that the interferon-induced antiviral factor IFITM3 inhibits SARS-CoV-2 infection by preventing SARS-CoV-2 spike-protein-mediated virus entry and cell-to-cell fusion. Analysis of a Chinese COVID-19 patient cohort demonstrates that the rs12252 CC genotype of IFITM3 is associated with SARS-CoV-2 infection risk in the studied cohort. These data suggest that individuals carrying the rs12252 C allele in the IFITM3 gene may be vulnerable to SARS-CoV-2 infection and thus may benefit from early medical intervention.

## 1. Introduction

The coronavirus disease 2019 (COVID-19) epidemic emerged in Wuhan, China, in December 2019, and has since spread worldwide. COVID-19 is caused by a new coronavirus, severe acute respiratory syndrome coronavirus 2 (SARS-CoV-2) [1,2]. Effective drugs and vaccines against SARS-CoV-2 are the hope of halting the rapid spread of COVID-19.

The severity of COVID-19 varies widely between infected individuals. Many are asymptomatic, some show mild symptoms, and a significant number of severe cases develop pneumonia, respiratory distress, and even death [3]. It is believed the polymorphisms of key genes in part underpin this broad range of COVID-19 symptoms, and some of these genes can be antiviral effectors that are induced by interferon (IFN), given the high sensitivity of SARS-CoV-2 to IFN inhibition [4]. Indeed, the study of the association between disease severity and single-nucleotide polymorphisms (SNPs) in IFN-stimulated genes (ISGs) has shed light on the critical roles of certain ISGs in host susceptibility to virus infections [5,6].

The interferon-induced transmembrane protein (IFITM) family has been demonstrated to possess broad antiviral activity by blocking virus entry [7,8,9,10,11,12,13,14]. IFITM3 was reported to inhibit respiratory viruses, including influenza A virus [7,8,9,15]. IFITM3 knockout mice succumb to the challenge of the otherwise non-lethal influenza A virus [16,17]. In humans, SNP rs12252-C of IFITM3 has been reported to be associated with disease severities in patients infected with 2009 H1N1 virus, H7N9 virus, Hantaan virus, and HIV-1 [18,19,20,21,22,23]. This rs12252 C SNP was proposed to generate an IFITM3 splice variant (ENST00000526811), which may lead to truncation of the N-terminal 21 amino acids of IFITM3 and thus the loss of antiviral activity [16,24]. Another SNP rs34481144-A, which is located in the promoter of IFITM3 and is associated with lower expression of IFITM3, was also linked to the disease severity of influenza virus infection [25].

However, the possible role of IFITM3 in the recent SARS-CoV-2 pandemic is controversial [26,27]. Here, we demonstrate that IFITM3 inhibits SARS-CoV-2 infection by preventing virus entry, and the first 21 amino acids of IFITM3 are indispensable for anti-SARS-CoV-2 activity. The analysis of a Chinese COVID-19 patient cohort indicates that the rs12252 CC genotype of IFITM3 is associated with the risk of acquiring SARS-CoV-2 infection and the weakened neutralizing antibody response.

## 2. Materials and Methods

### 2.1. Plasmids

The IFITM3 cDNA was cloned into the pQCXIP vector, and the Δ1–21, YLAA, Y20F, and Y20A mutations were generated with a PCR-based mutagenesis method as described previously [24,28]. The pCAGGS-SARS-CoV-2-S expression plasmid was a gift from He Huang (IPB, Beijing, China). The TMPRSS2 expression plasmid was purchased from Origene (Cat. No. RC208677). Transfection of plasmid DNA into cells was performed with Lipofectamine 2000 (Thermo Fisher Scientific, MA, USA) in accordance with the manufacturer’s instructions.

### 2.2. Pseudovirus Production and Infection

SARS-CoV-2 spike protein (S)-pseudoviruses were produced by transfecting HEK293T cells (CRL-2316) with psPAX2, pLenti-Luc/GFP, and pCAGGS-S. Forty-eight hours after transfection, the supernatant was collected and passed through a 0.45 μm filter. To determine viral infectivity, viruses were incubated with HEK293T-ACE2 cells for 48 h, and luciferase activities were measured using the Luciferase Assay System (Promega, WI, USA). Protein expression in cell lysates was detected by Western blotting.

### 2.3. β-Lactamase-Vpr Assay

BlaM-Vpr pseudoviral particle fusion assay was performed as previously described [29]. Briefly, cells were transfected with the following plasmids in a 10 cm dish, with 6 μg of psPAX2, 6 μg of BlaM-Vpr, and 6 μg of pCAGGS-S. Transfection was carried out using PEI. Forty-eight hours after transfection, the supernatant was collected and clarified with a 0.45 μm filter. Then, HEK293T-ACE2 cells were incubated with BlaM-Vpr pseudoviral particles for 8 h, before cells were washed with PBS and loaded with the CCF2-AM substrate (Invitrogen) for 2 h at 37 °C according to the manufacturer’s instructions. Following substrate loading, cells were washed and transferred to FACS tubes for flow cytometry analysis.

### 2.4. Cell–Cell Fusion Assay

Cell–cell fusion assay was performed as previously described [30]. In brief, HeLa cells transiently co-expressing SARS-CoV-2 S protein and HIV-1 Tat-Flag were co-cultivated with HEK293T target cells expressing ACE2 and HIV-1 LTR-Luc for 40 h. Cell fusion was quantified with luciferase assay based on HIV-1 Tat transactivated expression of luciferase.

### 2.5. Western Blotting

Cells were lysed in RIPA buffer (25 mM Tris /HCl (pH 7.4), 150 mM NaCl, 1% NP-40, 0.25% sodium deoxycholate, 1 mM EDTA). After clarification by centrifugation, equal amounts of cell lysates quantified by BCA™ Protein Assay Kit (Thermo Fisher Scientific, MA, USA) were separated in SDS-PAGE and transferred to nitrocellulose membranes. Then, membranes were probed with the indicated antibodies, anti-HA antibody from Sigma-Aldrich (St. Louis, MO, USA) (1:4000, Cat. No. H6908), anti-Flag antibody from Sigma-Aldrich (1:4000, Cat. No. F3165), anti-actin antibody from Sigma-Aldrich (1:5000, Cat. No. A1978), anti-SARS-CoV-2 N antibody from Sino Biological (1:1000, Cat. No. 40143-R019), and anti-IFITM3 antibody from Proteintech (1:1000, Cat. No. 11714-1-AP), followed by IRDye™ secondary antibodies (Odyssey, Lincoln, NE, USA). The signals were collected with the Odyssey Infrared Imaging System (Li-Cor, Lincoln, NE, USA).

### 2.6. Subjects and Samples

Samples of 203 patients (with a median age of 51, 105 males, and 98 females) diagnosed with quantitative RT-PCR for SARS-CoV-2 infection were investigated in this study. All patients required hospitalization in JinYinTan Hospital or FangCang Hospital, Wuhan. Throat swabs and plasma samples of patients were collected for additional viral testing, cytokine and chemokine quantification, and DNA extraction. This study was approved by the Ethical Review Board of the Institute of Pathogen Biology, Chinese Academy of Medical Sciences and Peking Union Medical College. Consent was obtained from competent patients directly or from relatives/friends/welfare attorneys of incapacitated patients.

### 2.7. Definition of Mild and Severe Cases

According to the new coronavirus disease-19 (COVID-19)-related pneumonia diagnosis and treatment guideline issued by the National Health Commission, patients with mild clinical symptoms and negative lung imaging present were defined as mild cases. Patients were defined as severe cases who had pneumonia confirmed by chest imaging and had an oxygen saturation (SaO_2_) of 94% or less while they were breathing ambient air or a ratio of the partial pressure of oxygen (PaO_2_) to the fraction of inspired oxygen (FiO_2_) (PaO_2_:FiO_2_) at or below 300 mm Hg.

### 2.8. Sequencing and Genotyping of rs12252

The region encompassing the human IFITM3 rs12252 sequences was amplified by PCR from DNA obtained from throat swabs. The amplification was performed using the following forward and reverse primers, 5′-GGAAACTGTTGAGAAACCGAA-3′ and 5′-CATACGCACCTTCACGGAGT-3′ (Beijing Institute of Genomics, Shenzhen, China). The products encompassing IFITM3 rs12252 were purified and Sanger sequenced on an Applied Biosystems 3730 × 1 DNA Analyzer (GATC Biotech, Konstanz, Germany). SNP genotypes were identified by MEGA-X. Homozygotes were called based on high, single-base peaks with high Phred quality scores, while heterozygotes were identified based on low, overlapping peaks of two bases with lower Phred quality scores relative to surrounding base calls. SNP genotype frequencies in our sequencing were compared with that of the Chinese Han population from the 1000 Genomes Project database.

### 2.9. Cytokines and Chemokines Quantification

Plasma cytokines and chemokines were measured using the Human Cytokine Standard 27-Plex Assays panel and the Bio-Plex 200 system (Bio-Rad, CA, USA) according to the manufacturer’s instructions. Plasma samples from four healthy adults were used as controls for cross-comparison. These four healthy adults were tested negative for SARS-CoV-2 by RT-PCR and did not have respiratory symptoms or other health-related complaints before sample collection.

### 2.10. SARS-CoV-2 Viral RNA Analysis

SARS-CoV-2 viral RNA content was tested using validated primers and probes targeting the nucleocapsid (N) gene, and data were acquired with the Bio-Rad real-time PCR system [31].

### 2.11. Enzyme-Linked Immunosorbent Assay

Enzyme-linked immunosorbent assay (ELISA) was used for detecting IgM and IgG antibodies against the SARS-CoV-2 N, S, and receptor-binding domain (RBD) as previously described [32]. Briefly, the purified RBD, S, and N proteins of SARS-CoV-2 were used as coating antigens. Horseradish peroxidase (HRP)-conjugated goat anti-human IgG (Sigma-Aldrich, St. Louis, MO, USA) and Horseradish Peroxidase-conjugated AffiniPure Goat Anti-Human IgM (Jackson ImmunoResearch Inc., West Grove, PA, USA) were used as the second antibody in 1:60,000 dilutions. The optimal coating concentrations of RBD, S, and N antigen were 10 ng/well, 20 ng/well, and 10 ng/well. The optimal plasma dilutions were 1:400.

### 2.12. Neutralizing Antibody Detection

Among the 203 patient samples in this cohort, we had sufficient plasma samples of only 81 patients to measure the neutralizing antibody level. The neutralization assay was performed with Vero E6 cells. Serial two-fold dilutions of plasma samples (starting at 1:10) were pre-incubated with SARS-CoV-2 at 100 TCID50 (50% tissue culture infective doses) for 2 h and then added to Vero E6 cells. The virus/plasma mixtures were removed after 1 h, and fresh growth medium was added to each well. The cytopathic effects were evaluated 5 days after incubation at 37 °C. For each plasma dilution, 4 duplicate wells were used. The neutralizing antibody titers were calculated using the Reed and Muench method [33].

### 2.13. Statistical Analysis

Statistical analysis of genetic data was performed using the chi-square test with SPSS software (version 23.0, SPSS Inc., Chicago, IL, USA). The statistical significance of allele frequencies was determined with Fisher’s exact test. Statistical analysis of cytokine and chemokine data and antibody data was performed using Student’s *t* test or the Mann–Whitney test, and the correlation analysis was performed using Pearson or Spearman correlation analysis with SPSS software (version 23.0). * indicates a significant difference *p* < 0.05, ** indicates *p* < 0.01, *** indicates *p* < 0.001, and **** indicates *p* < 0.0001.

## 3. Results

### 3.1. IFITM3 Restricts SARS-CoV-2 Infection

**Figure 1:**
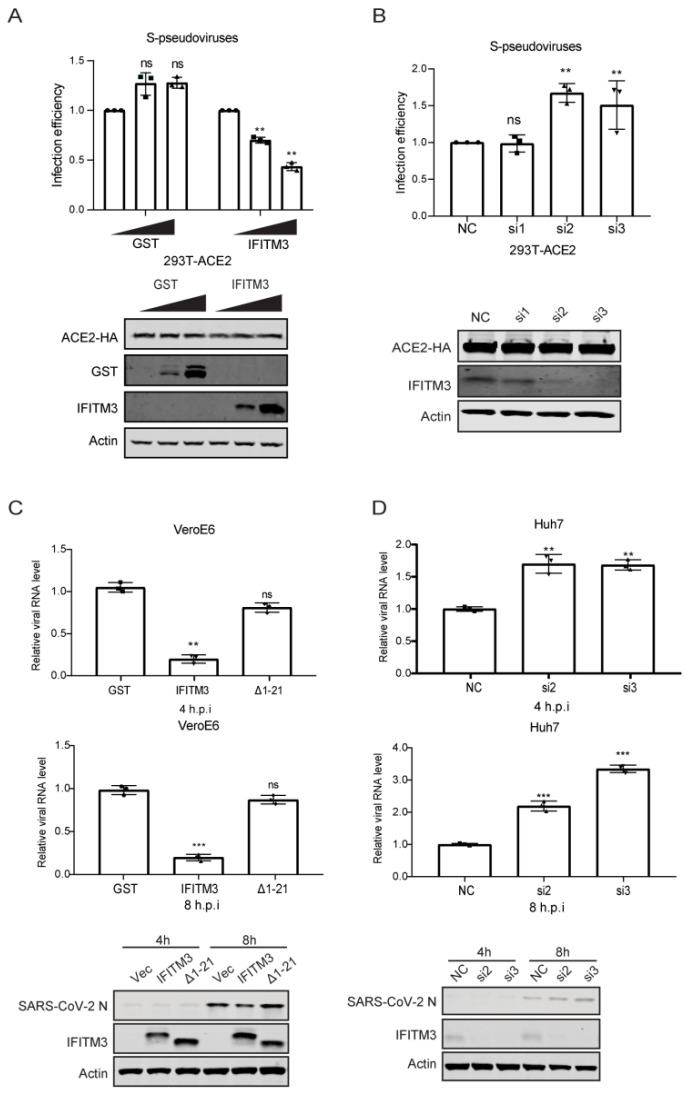
IFITM3 inhibits SARS-CoV-2 infection. (**A**) HEK293T-ACE2 cells were transfected with increasing amounts of GST or IFITM3 plasmid DNA and then infected with lentiviral reporter viruses pseudotyped with SARS-CoV-2 spike protein. Infection efficiency was determined by luciferase activities and normalized to that of vector DNA-transfected cells. Statistical significance was determined with Student’s *t* test. ns, not significant; **, *p* < 0.01. Expression of GST, IFITM3, ACE2, and actin was measured by Western blotting. (**B**) HEK293T-ACE2 cells were transfected with siRNAs targeting IFITM3 (si1, si2, si3) and then infected with lentiviral reporter viruses pseudotyped with SARS-CoV-2 spike protein. Infection efficiency was determined by luciferase activities and normalized to that of cells transfected with control siRNA (NC). Expression of IFITM3, ACE2, and actin was measured by Western blotting. (**C**) Vero E6 cells were transfected with GST, IFITM3, or Δ(1–21) IFITM3 and then infected with SARS-CoV-2 for 4 h or 8 h. Viral RNA level was determined by qRT-PCR, and expression of N, IFITM3, and actin was measured by Western blotting. (**D**) Huh7 cells were transfected with siRNAs targeting IFITM3 (si1, si2, si3) and then infected with SARS-CoV-2 for 4 h or 8 h. Viral RNA level was determined by qRT-PCR, and expression of N, IFITM3, and actin was measured by Western blotting. Data in the bar charts are presented as mean ± SD of three independent experiments. ns, not significant; NC, negative control; si1, IFITM3 siRNA1; si2, IFITM3 siRNA2; si3, IFITM3 siRNA3; h.p.i., hour post-infection. ns, not significant; **, *p* < 0.01; ***, *p* < 0.001.

We and others reported that many IFN-inducible genes, including IFITM3, were upregulated in COVID-19 patients [34,35,36]. Given the broad antiviral activity of IFITM3 [7,8,9,10,11], we investigated whether IFITM3 inhibits SARS-CoV-2 infection. Since IFITM3 impedes virus entry, we measured the effect of IFITM3 on the infection of lentiviral particles pseudotyped with SARS-CoV-2 spike protein. IFITM3 overexpression diminished SARS-CoV-2 S-mediated infection by 2-fold (Figure 1A). Then, siRNAs targeting IFITM3 led to a 50% increase in the infection of SARS-CoV-2 S-pseudotyped virus (Figure 1B). The N-terminal 21 amino acids of IFITM3, which contain the endocytic sorting signal 20-YEML-23, were reported to regulate IFITM3 trafficking in cells and antiviral activity [14,24,28]. The N-terminal 21 amino acids, the tyrosine-based protein sorting signal 20-YEML-23 in particular, enable IFITM3 to undergo endocytosis and are essential for the antiviral function of IFITM3 [24,28]. We have thus generated the N-terminal 21 amino acids deletion mutant (Δ1–21), the endocytic sorting signal mutants YLAA, Y20F, and Y20A, as controls of non-antiviral IFITM3 mutants. Not surprisingly, the N-terminal 21 amino acids deletion mutant (Δ1–21), the endocytic sorting signal mutant YLAA, Y20F, and Y20A, all lost the ability to inhibit the infection of SARS-CoV-2 S pseudoviruses (Figure S1). Next, we examined the effect of IFITM3 on SARS-CoV-2 infection of Vero E6 and Huh 7 cells. IFITM3 transfected Vero E6 cells were infected with SARS-CoV-2. Viral RNA was quantitated by qRT-PCR, and a 5-fold decrease was observed in IFITM3-transfected cells at both 4 hpi and 8 hpi (Figure 1C). Consistent with its neutral effect on SARS-CoV-2 S pseudoviruses, the N-terminal 21 amino acids deletion mutant (Δ1–21) did not inhibit SARS-CoV-2 infection (Figure 1C). In IFITM3-knockdown Huh 7 cells, levels of SARS-CoV-2 viral RNA and N protein expression increased by 2-fold (Figure 1D). These data demonstrate the anti-SARS-CoV-2 activity of IFITM3.

### 3.2. IFITM3 Inhibits SARS-CoV-2 Entry

**Figure 2:**
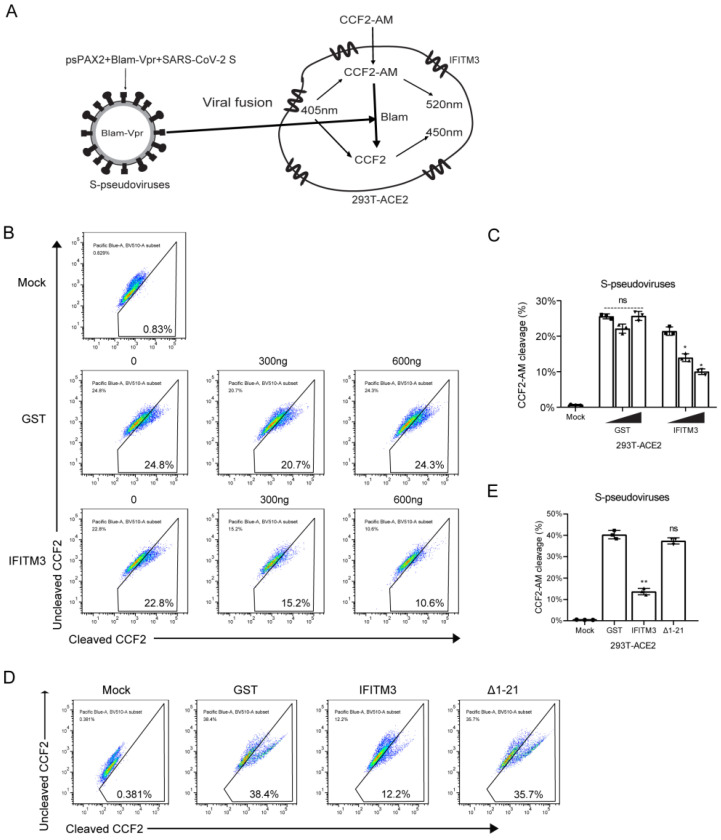
IFITM3 inhibits SARS-CoV-2 entry. (**A**) Illustration of the β-lactamase-Vpr assay. (**B**) HEK293T-ACE2 cells were transfected with increasing amounts of GST or IFITM3 and then infected with BlaM pseudovirus particles. BlaM activity was determined by flow cytometry. (**C**) Summary of the percentages of cells exhibiting CCF2-AM cleavage in (**B**). (**D**) HEK293T-ACE2 cells were transfected with IFITM3 or Δ(1–21) IFITM3 DNA, and then infected with BlaM pseudovirus particles. BlaM activity was determined by flow cytometry. (**E**) Summary of the percentages of cells exhibiting CCF2-AM cleavage in (**D**). Data in the bar charts are presented as mean ± SD of three independent experiments. ns, not significant. *, *p* < 0.05; **, *p* < 0.01.

As IFITM proteins diminish the entry of different viruses, we next sought to examine the effect of IFITM3 on SARS-CoV-2 entry by BlaM-Vpr fusion assay. BlaM-Vpr is incorporated into SARS-CoV-2 S-pseudotyped lentiviral particles and delivered into target cells upon virus entry. Cleavage of CCF2-AM by BlaM-Vpr produces a fluorescence emission shift from green (520 nm) to blue (450 nm) (Figure 2A). As shown in Figure 2B,C, IFITM3 expression reduced SARS-CoV-2 S-mediated virus entry in a dose-dependent manner. Compared with wild-type IFITM3, IFITM3(Δ1–21) had minimal impact on virus entry (Figure 2D,E). These data support that IFITM3 inhibits SARS-CoV-2 infection by preventing virus entry.

We further investigated the effect of IFITM3 on SARS-CoV-2 S-induced membrane fusion in the cell–cell fusion assay. SARS-CoV-2 S and Tat transfected HeLa cells were co-cultured with HEK293T-ACE2 cells that expressed IFITM3 and Tat-inducible LTR-luciferase. After cell–cell fusion, Tat transactivates luciferase expression. Results of luciferase assay showed that IFITM3 inhibited SARS-CoV-2 S-mediated cell–cell fusion, while no effect was observed for IFITM3(Δ1–21) (Figure S2). Together, these data demonstrate that IFITM3 inhibits SARS-CoV-2 S-protein-mediated virion–cell fusion, as well as cell–cell fusion.

### 3.3. TMPRSS2 Attenuated the Inhibition of IFITM3

**Figure 3:**
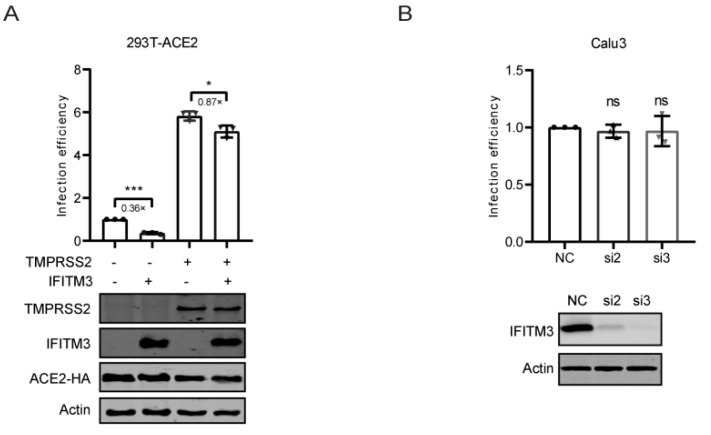
TMPRSS2 attenuated the inhibition of IFITM3. (**A**) HEK293T-ACE2 cells were transfected with IFITM3, TMPRSS2, or IFITM3 together with TMPRSS2 plasmid DNAs and then infected with lentiviral reporter viruses pseudotyped with SARS-CoV-2 spike protein. Infection efficiency was determined by luciferase activities and normalized to that of vector DNA-transfected cells. Expression of TMPRSS2, IFITM3, ACE2, and actin was measured by Western blotting. (**B**) Calu3 cells were transfected with siRNAs targeting IFITM3 (si2, si3) and then infected with lentiviral reporter viruses pseudotyped with SARS-CoV-2 spike protein. Infection efficiency was determined by luciferase activities and normalized to that of cells transfected with control siRNA (NC). Expression of IFITM3 and actin was measured by Western blotting. Data in the bar charts are presented as mean ± SD of three independent experiments. Statistical significance was determined with Student’s *t* test. ns, not significant; *, *p* < 0.05; ***, *p* < 0.001; NC, negative control; si2, IFITM3 siRNA2; si3, IFITM3 siRNA3.

TMPRSS2 allows SARS-CoV-2 entry at the cell surface [37,38], and has been shown to render SARS-CoV-2 resistant to the inhibition of IFITM3 [26,39,40]. Indeed, when TMPRSS2 and IFITM3 were co-expressed in 293T-ACE2 cells, infection of SARS-CoV-2 S-pseudotyped viruses was less inhibited by IFITM3 compared to that in cells not expressing TMPRSS2 (Figure 3A). We further knocked down IFITM3 in TMPRSS2-positive Calu-3 cells, and did not observe any effect on infection of SARS-CoV-2 S-pseudotyped virus (Figure 3B). These data are consistent with previous reports that TMPRSS2 counters the inhibition of IFITM proteins [26,39,40].

### 3.4. The rs12252 CC Genotype of IFITM3 Is Associated with the Risk of Acquiring SARS-CoV-2 Infection

**Figure 4:**
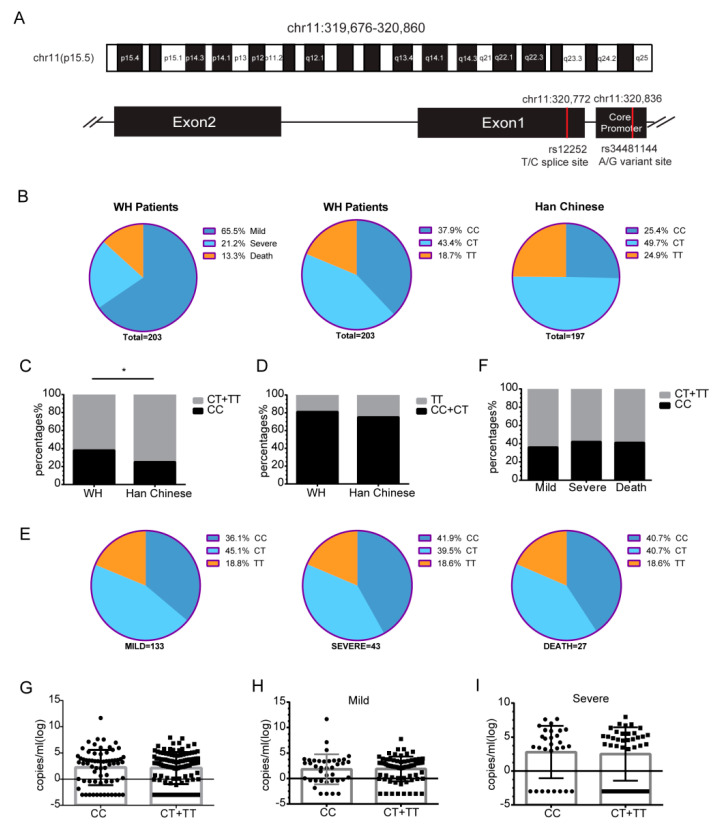
The rs12252 CC genotype of IFITM3 is associated with the risk of SARS-CoV-2 infection. (**A**) Illustration of SNPs rs12252 and rs34481144 of IFITM3. (**B**) Frequency of rs12252 alleles in our 203 COVID-19 patient cohort (WH patients), including 133 mild cases (65.5%), 43 severe cases (21.2%), and 27 deaths (13.3%). Han Chinese: healthy Chinese Han population (from the 1000 genome project). (**C**) The frequency of the CC genotype (37.9%) was increased (*p* = 0.04) in our COVID-19 patient cohort. (**D**) Frequency of the rs12252-C allele in our cohort and in the Chinese Han population (data from the 1000 genomes project). (**E**) Frequencies of the rs12252 alleles in mild, severe, and death cases. (**F**) Frequency of the CC genotype in mild, severe, and death cases. (**G**) Viral load in the throat swab samples from the CC and CT/TT carriers. (**H**,**I**) Viral load in the CC and CT/TT carriers from the mild group (**H**) and the severe group (**I**). WH patients, Wuhan patients; *, *p* < 0.05.

Two SNPs of IFITM3, rs12252-C, and rs34481144-A (Figure 4A) were reported to be associated with the disease severity of several virus infections [18,19,20,21,22,23]. To investigate the possible association of these SNPs with SARS-CoV-2 infection, we sequenced the IFITM3 locus of 203 hospitalized COVID-19 patients, which included 133 mild cases (65.5%), 43 severe cases (21.2%), and 27 deaths (13.3%) (Figure 4B). Sequence analysis showed that 77 patients (37.9%) had the rs12252 CC genotype, 88 patients (43.4%) had the CT genotype, and 38 patients (18.7%) had the TT WT genotype (Figure 4B). Compared with the prevalence rate of 25.4% CC in the healthy Chinese Han population (from the 1000 genome project), the frequency of the CC genotype in 203 COVID-19 patients (37.9%) was significantly increased (*p* = 0.04) (Figure 4B,C), even though total C allele frequency did not show significant changes (Figure 4D). This result suggests that the CC genotype may increase the risk of SARS-CoV-2 infection. We further explored the correlation between CC genotype and disease severity, and found that the distributions of CC and CT/TT were 36.1% and 63.9% in mild cases, 41.9% and 58.1% in severe cases, 40.7% and 59.3% in deaths (Figure 4E,F). Although no statistically significant differences between these groups were found, the CC genotype in the severe and dead population tends to be over-presented. Next, the viral loads in throat swabs were determined, and the results showed no differences between CC and CT/TT genotypes in the entire patient cohort (Figure 4G), patients with mild symptoms (Figure 4H), or patients with severe symptoms (Figure 4I).

Furthermore, we investigated the association of another SNP rs34481144-A with SARS-CoV-2 infection. Only one patient in our cohort carries the AG genotype, which was consistent with 1000 genomes project data. Due to the limited number of the AG genotype in our patient cohort, we could not conclude the association of rs34481144 A with SARS-CoV-2 infection (Table S1).

### 3.5. Levels of Cytokines and Chemokines in COVID-19 Patients with CC or CT/TT Genotypes

**Figure 5:**
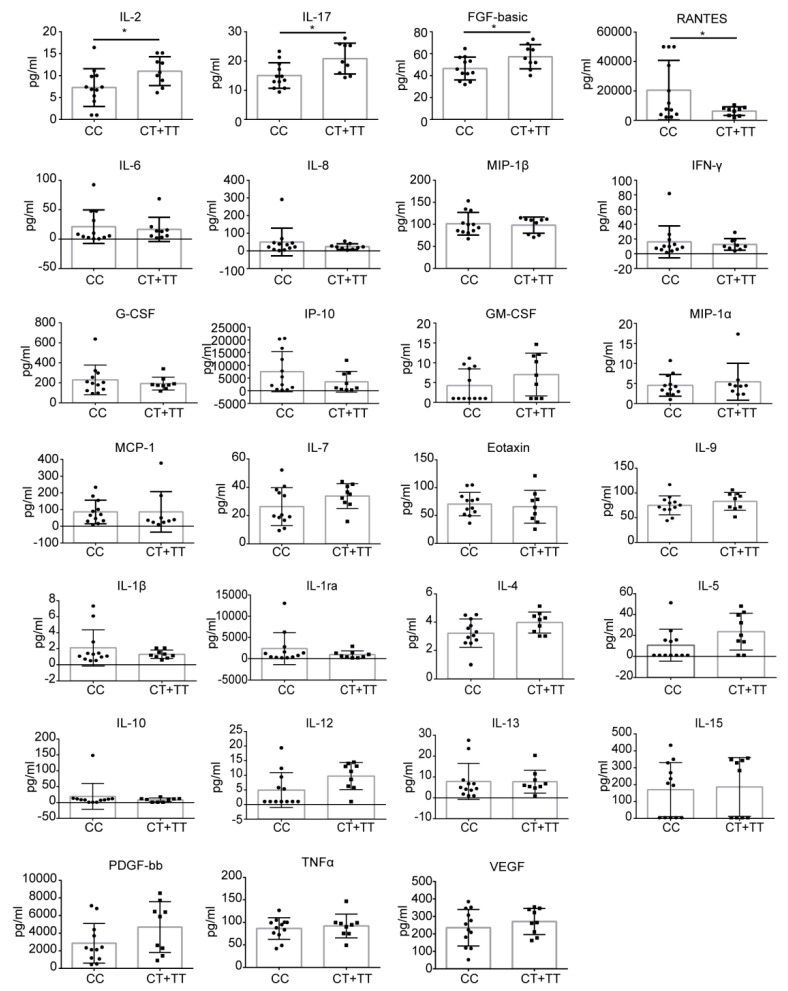
Levels of cytokines and chemokines in COVID-19 patients with the CC genotype or the CT/TT genotypes. Statistically significant differences were observed for IL-2 (*p* = 0.043), IL-17 (*p* = 0.013), FGF-basic (*p* = 0.034), and RANTES (*p* = 0.034). *, *p* < 0.05.

Cytokine and chemokine levels in 21 patients (12 CC, 7 CT, and 2 TT genotypes) were determined. Compared with the healthy controls, plasma levels of IL-1β, IL1ra, IL-2, IL-4, IL-6, IL-8, IL-7, IL-9, IL-13, IL-15, IL-17, basic FGF, IP-10, MCP-1, MIP-1α, PDGF, VEGF, MIP-1β, TNF-α, and RANTES were significantly elevated in COVID-19-related pneumonia patients, whereas levels of IL-5, IL-10, IL-12, Eotaxin, G-CSF, GM-CSF, and IFN-γ were similar between the healthy control group and COVID-19 patients (Figure S3A). Further comparison of mild and severe cases showed that levels of MCP-1, MIP-1β, and MIP-1α were significantly higher in severe cases (Figure S3B). We also found that patients with the CC genotype had lower levels of IL-2, IL-17, and basic FGF than patients with the CT/TT genotype, but the level of RANTES was higher in CC patients, while levels of IL-1β, IL1ra, IL-4, IL-6, IL-8, IL-7, IL-9, IL-13, IL-15, IP-10, MCP-1, MIP-1α, PDGF, VEGF, MIP-1β, TNF-α, IL-5, IL-10, IL-12, Eotaxin, G-CSF, GM-CSF, and IFN-γ did not show a statistically significant difference between these two groups (Figure 5).

### 3.6. The rs12252 CC Genotype Might Be Associated with SARS-CoV-2 Neutralizing Antibody Positivity

**Figure 6:**
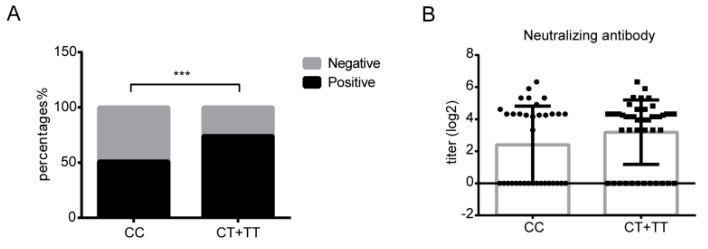
The rs12252 CC genotype is associated with lower positivity frequency of SARS-CoV-2 neutralizing antibody. (**A**) The positivity frequency of neutralizing antibodies in patients with different rs12252 genotypes in two to three weeks after symptom onset (*p* = 0.0008). (**B**) Neutralizing antibody titers in CC and CT/TT patients. ***, *p* < 0.001.

We next measured SARS-CoV-2 neutralizing antibodies in 81 patients (35 CC, 32 CT, and 14 TT genotypes) two to three weeks after the onset of symptoms. We found that 18 of 35 patients with the CC genotype (51.4%) were positive for neutralizing antibodies, and 34 were positive (73.9%) in the 46 patients of the CT or TT genotype (Figure 6A). The neutralizing antibody positivity rate was significantly lower in the CC group (χ^2^ = 4.356, *p* = 0.037), although no difference in neutralizing antibody titers in all positive cases was observed (Figure 6B). These data suggest that the rs12252 CC genotype is probably associated with poor neutralizing antibody production. In addition, we measured the levels of anti-N antibody, anti-S antibody, and anti-receptor-binding-domain (RBD) antibody. There were no differences in the levels of anti-N antibody or anti-S antibody between patients with CC and CT/TT genotypes, whereas patients with the CC genotype had a lower level of anti-RBD antibody IgG (Figure S4).

## 4. Discussion

IFITM3 has been reported to inhibit a number of pathogenic viruses in the families of orthomyxoviruses, flaviviruses, filoviruses, and coronaviruses [7,8,9,10,11,12,13,14]. Interestingly, IFITM3 was reported to restrict the entry mediated by the spike proteins of SARS-CoV, MERS-CoV, hCoV-229E, and hCoV-NL63, yet it enhances the infection of hCoV-OC43 [8,9,15,41]. IFITM1, IFITM2, and IFITM3 were reported to inhibit SARS-CoV-2 infection, particularly in cells not expressing the TMPRSS2 protease, which allows SARS-CoV-2 entry at the plasma membrane [26,39,40,42,43]. In this study, we further showed that IFITM3 inhibited SARS-CoV-2 infection by preventing virus entry. The first 21 amino acids of IFITM3, which carry the endocytic sorting signal 20YEML23 and the Y20 phosphorylation site and have been shown essential for the restriction of VSV and influenza A virus, are also important for inhibiting SARS-CoV-2. In addition, results of BlaM-Vpr fusion assay and cell–cell fusion assay support that IFITM3 inhibited SARS-CoV-2 S-protein-mediated virion–cell fusion and cell–cell fusion, consistent with a previous report [40].

The rs12252 C allele of IFITM3 has been reported to be associated with the disease severity of several viral infections, including influenza virus, Hantaan virus, and HIV-1 [18,19,20,21,22,23]. We now show that our COVID-19 cohort has a significantly higher presentation of the rs12252 CC homozygous genotype compared to the general control population, which is in agreement with previous reports [44,45,46]. The rs12252 C allele was predicted to affect the splicing of IFITM3 RNA, resulting in the loss of the first 21 amino acids of IFITM3 and the expression of a truncated version of IFITM3 protein Δ(1–21), which did not inhibit influenza A virus (A/WSN/1933) [16]. Our study showed that this Δ(1–21) IFITM3 mutant also lost the ability to inhibit SARS-CoV-2 infection, which at least partially underlies the increased risk of acquiring SARS-CoV-2 for those carrying the rs12252 CC genotype. Given the current lack of strong evidence supporting the expression of Δ(1–21) IFITM3 in the rs12252 CC carriers [47,48], the molecular mechanisms behind the association of rs12252 CC with viral infections warrant further investigation. In any case, the rs12252 C may serve as one genetic marker for vulnerability to SARS-CoV-2 infection. IFITM3 SNP rs12252 has been reported to be associated with SARS-CoV-2 infection risk, COVID-19 disease outcome, and mortality rate in some cohorts [44,45,46,49,50,51], but not in others [52,53]. In this cohort of Chinese COVID-19 patients, we did not observe a statistically significant difference in the frequency of the CC genotype between the mild, severe, and fatal COVID-19 cases, nor the viral loads, but did find significantly higher presentations of the CC genotype in SARS-CoV-2 positive individuals, suggesting an association of IFITM3 with the acquisition of SARS-CoV-2 infection. These results together suggest that the ultimate effect of IFITM3 polymorphisms on SARS-CoV-2 infection and COVID-19 disease severity might depend on the genetic background of the studied populations.

IFITM3 SNP rs34481144 was reported to be associated with the risk for hospitalization after SARS-CoV-2 infection [50,51]. In this study, we also investigated the SNP rs34481144 at the IFITM3 core promoter, and found only one patient carrying the AG genotype in our cohort. This is consistent with 1000 genomes project data, i.e., Asian descents have a very low frequency of rs34481144 AA and AG genotypes, which precludes the further study of the possible association of this SNP with COVID-19 using our cohort.

COVID-19 is characterized by high levels of pro-inflammatory cytokines and chemokines, including IL-2, IL-7, IL-10, G-CSF, IP10, MIP-1A, MCP-1, and TNFα, especially in patients cared for in ICU [3]. Early studies have shown that increased levels of pro-inflammatory cytokines in plasma, such as MCP1, were associated with pulmonary inflammation and severe lung damage in SARS-CoV patients [54]. In our COVID-19 cohort, we observed increased levels of IL-2, IL-4, IL-15, IL17, and IP-10, which likely activate both T-helper-1 (Th1) and T-helper-2 (Th2) cells and lead to lung inflammation and damage, similar to what has been reported in SARS-CoV and MERS-CoV infections. In addition, we detected higher levels of RANTES and lower levels of IL-2, IL17, and basic FGF in CC carriers, which suggests that Th2 and T-helper-17 (Th17) cell functions might have been compromised in the CC patients. A detailed follow up of cytokine levels in COVID-19 patients at all stages of the disease will help to illustrate a more accurate map of host inflammatory responses to SARS-CoV-2 infection. Only 21 patients had sequential samples, and we therefore chose to evaluate the levels of cytokines and chemokines in these patients. Moreover, for the reason of strict blood sample control, we did not obtain sufficient samples to measure the levels of cytokines and chemokines in some patients. Due to the enormous pressures doctors and nurses faced in the early phase of the COVID-19 pandemic, we were unable to have regular periods of sample collection nor detailed clinical information, which limits the further interpretation of our data on cytokine and chemokine levels and association with IFITM3 polymorphisms in the context of clinical characteristics other than disease severity.

Polymorphisms in IFITM3 have been reported to modulate antibody responses to virus infection. Individuals with the rs12252 CC genotype have been shown to have higher pre-existing immunity to pdm09H1N1 than those with the TT genotype in a young people cohort [55]. Another study reported that the CC carriers presented lower seroconversion (SCR) and HI antibody titer 28 days after inoculation of the trivalent inactivated vaccine (TIV) [56]. In our study, we found that patients with the CC genotype had a lower positivity frequency of neutralizing antibody and lower levels of RBD antibody IgG than that in patients with CT/TT genotypes (Figure S4). These findings indicate that the rs12252 SNP in IFITM3 may modulate antibody responses, especially IgG production. A previous study showed that IFITM3 deficient mice had reduced the number of activated B cells and dysfunction of the germinal center to plasma/memory transition [56], which may in part explain the association of deficient antibody response with the rs12252 SNP in IFITM3.

In summary, our data demonstrate that IFITM3 is able to inhibit SARS-CoV-2 by impeding virus entry. We further observed the association of the rs12252 SNP in IFITM3 with a higher risk of SARS-CoV-2 acquisition, thus revealing one genetic factor underpinning the susceptibility to COVID-19.

## Data Availability

The data presented in this study are available upon request from the corresponding author.

## Supplementary Materials

The following supporting information can be downloaded at: https://www.mdpi.com/article/10.3390/v14112553/s1, Figure S1: Deletion of first 21 aa of IFITM3 abolishes its antiviral activity; Figure S2: IFITM3 inhibits SARS-CoV-2 spike-protein-mediated cell–cell fusion; Figure S3: Levels of cytokines and chemokines in COVID-19 patients; Figure S4: Levels of anti-N, anti-S and anti-RBD antibodies in COVID-19 patients with the CC and CT/TT genotypes; Table S1: Genotype frequencies of IFITM3 rs34481144.
