# Supplementary material for: IFITM3 Inhibits SARS-CoV-2 Infection and Is Associated with COVID-19 Susceptibility
Source: Viruses. 2022 Nov 18;14(11):2553. doi: 10.3390/v14112553 (PMC9692367; doi:10.3390/v14112553)
Supplement: Supplementary file 1 [file viruses-14-02553-s001.zip › viruses-1968511-supplementary.pdf]

Supplementary Material

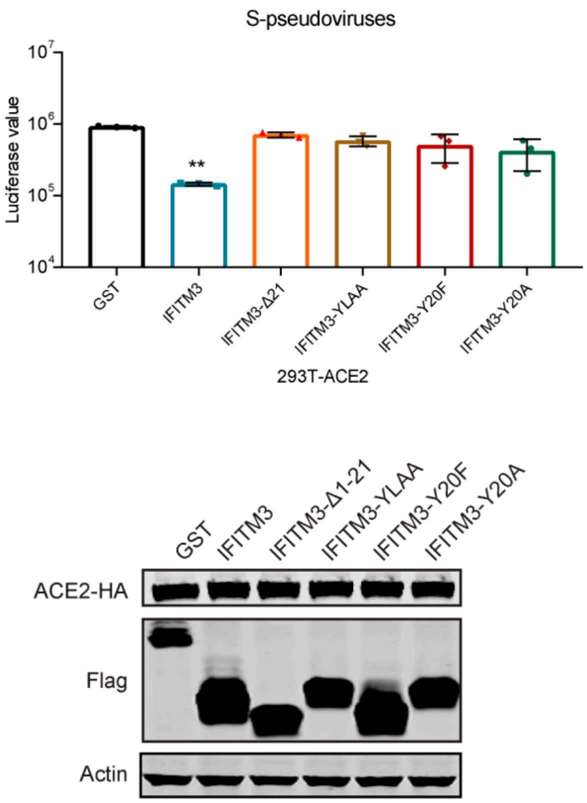

Figure S1. Deletion of first 21 aa of IFITM3 abolishes its antiviral activity. HEK293T-ACE2 cells were transfected with GST, IFITM3, and IFITM3 mutants Δ(1-21), YLAA, Y20F, or Y20A, and then infected with lentiviral reporter viruses pseudotyped with SARS-CoV-2 spike protein. Infection efficiency was determined by luciferase activities. Data in the bar charts are mean ± SD of 3 independent experiments. Statistical significance was determined with the Student 's *t* test. **, $p < 0.01$. Expression of GST, IFITM3, ACE2 and actin was measured by Western blotting.

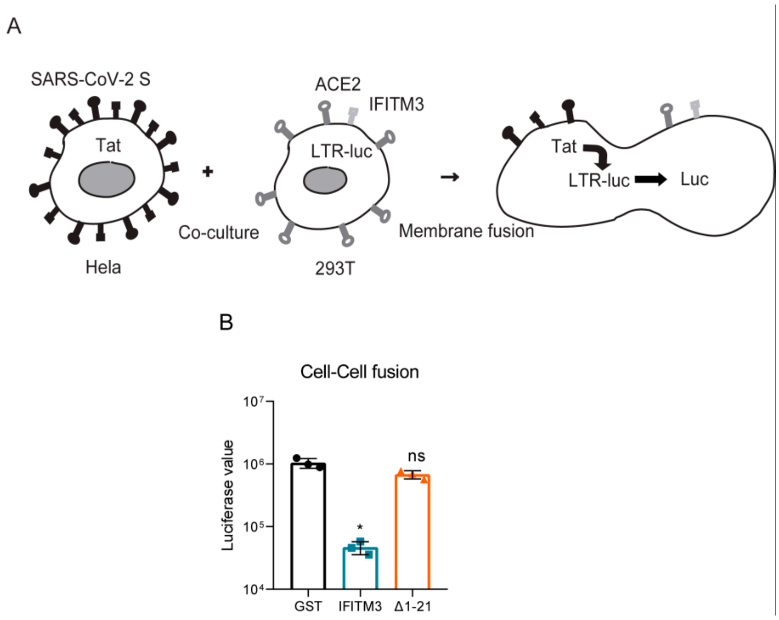

Figure S2. IFITM3 inhibits SARS-CoV-2 spike protein-mediated cell-cell fusion. **(A)** Illustration of the cell-cell fusion assay. **(B)** HeLa cells transiently expressing SARS-CoV-2 S protein and HIV-1 Tat protein were co-cultivated with HEK293T-ACE2 target cells expressing IFITM3 and HIV-1 LTR-luc. After 40 h of co-culture, cell-cell fusion was determined by measuring luciferase activity. Data in the bar charts are mean±SD of 3 independent experiments. ns, not significant; *, $p < 0.05$.

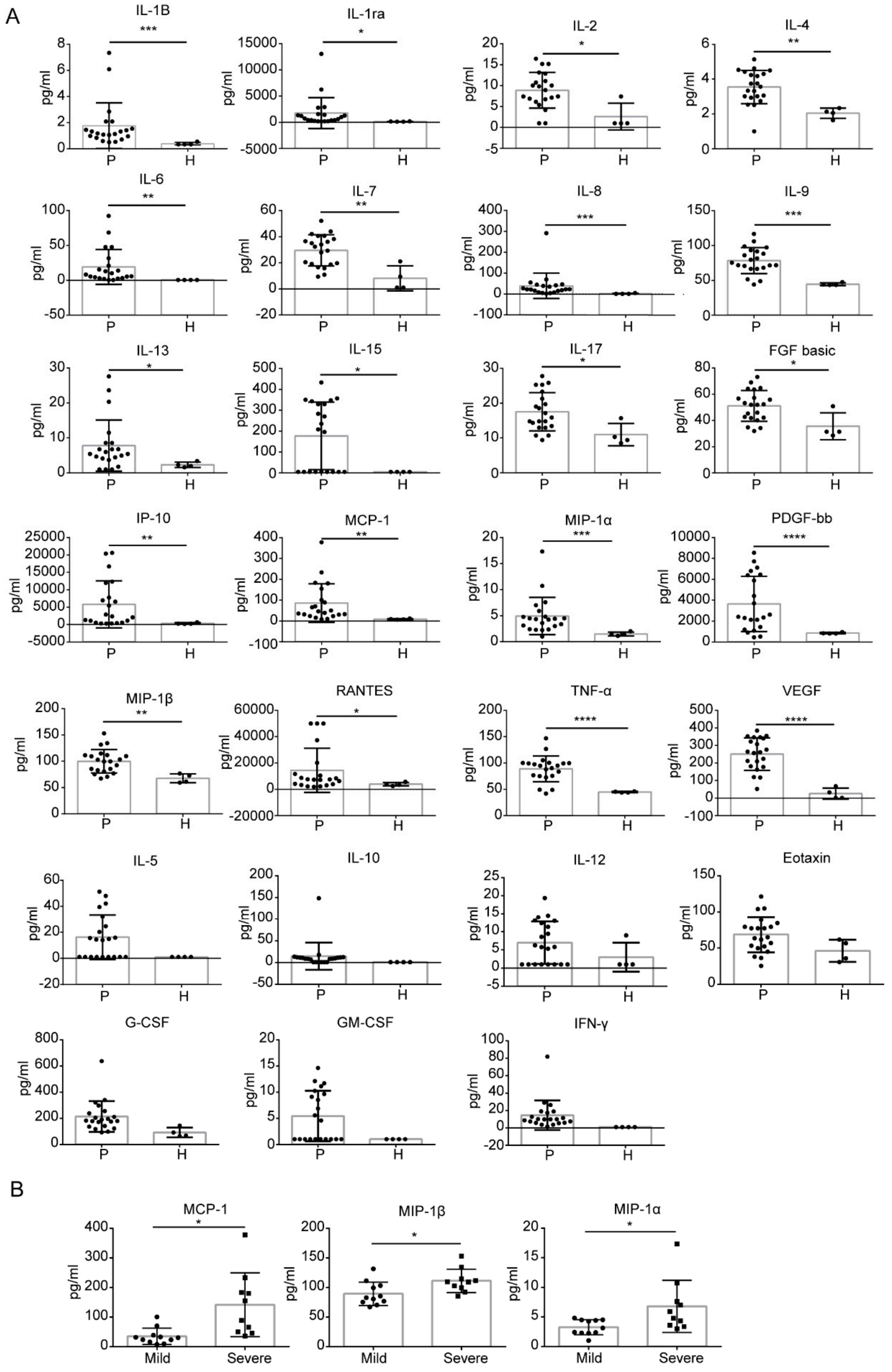
A
IL-1B
***
pg/ml
P
H
IL-1ra
*
IL-2
*
IL-4
**
IL-6
**
IL-7
**
IL-8
***
IL-9
***
IL-13
*
IL-15
*
IL-17
*
FGF basic
*
IP-10
**
MCP-1
**
MIP-1α
***
PDGF-bb
****
MIP-1β
**
RANTES
*
TNF-α
****
VEGF
****
IL-5
IL-10
IL-12
Eotaxin
G-CSF
GM-CSF
IFN-γ
B
MCP-1
*
MIP-1β
*
MIP-1α
*
Mild
Severe

Figure S3. Levels of cytokines and chemokines in COVID-19 patients. **(A)** Cytokine levels in COVID-19 patients and healthy individuals. Statistically significant differences were observed for IL-1β (p =0.006), IL-1ra (p =0.022), IL-2 (p =0.011), IL-4 (p =0.005), IL-6 (p =0.002), IL-7 (p = 0.003), IL-8 (p =0.0006), IL-9 (p =0.0004), IL-13 (p =0.045), IL-15 (p =0.047), IL-17 (p =0.032), FGF basic (p =0.022), IP-10 (p =0.004), MCP-1 (p =0.001), MIP-1α (p =0.0003), PDGF ($p<$ 0.0001), MIP-1β (p =0.009), RANTES (p =0.010), TNF-α ($p<0.0001$), VEGF ($p<0.0001$). **(B)** Plasma levels of MCP-1, MIP-1β, and MIP-1α in mild and severe COVID-19 cases. Statistically significant differences were observed for MCP-1 (p =0.012), MIP-1β (p =0.021), MIP-1α (p = 0.035). P, patients; H, healthy individuals; *, $p < 0.05$; **, $p < 0.01$; ***, $p < 0.001$; ****, $p <$ 0.0001.

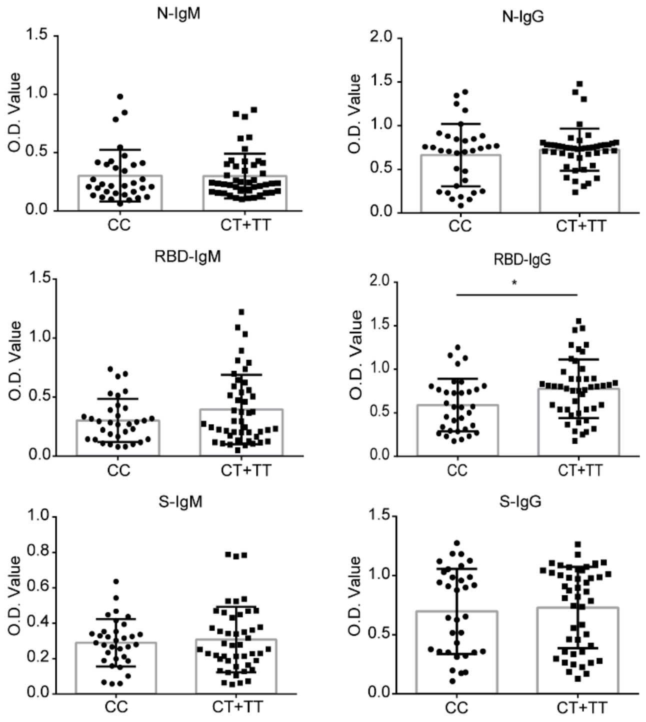

Figure S4. Levels of anti-N, anti-S and anti-RBD antibodies in COVID-19 patients with the CC and CT/TT genotypes. Levels of RBD-IgG are lower in patients with the homozygous CC genotype (RBD-IgG, p =0.013). *, $p < 0.05$.

Table S1. Genotype frequencies of IFITM3 rs34481144.

Genotype	All patients (n=203)	Han Chinese (n=211)	British in England and Scotland (n=92)
GG	202(99.5%)	209(99.1%)	15(16.3%)
GA	1(0.5%)	2(0.9%)	50(54.4%)
AA	0(0%)	0(0%)	27(29.3%)
